# Preservation and sterilization methods of the meniscal allografts: literature review

**DOI:** 10.1007/s10561-013-9396-7

**Published:** 2013-09-03

**Authors:** Patrycja Mickiewicz, Marcin Binkowski, Henryk Bursig, Zygmunt Wróbel

**Affiliations:** 1 X-ray Microtomography Lab, Department of Biomedical Computer Systems, Institute of Computer Science, Faculty of Computer and Material Science, University of Silesia, ul. 75 Pułku Piechoty 1, Budynek H, Segment C, P8, 41-500 Chorzów, Poland; 2 Regional Blood Center, Tissue Bank Katowice, Raciborska 15, 40-074 Katowice, Poland

**Keywords:** Meniscal allograft preservation, Meniscal allograft sterilization, Cryopreservation, Deep-freezing

## Abstract

Nowadays, there are four types of meniscal allografts known: fresh, cryopreserved, deep-frozen and lyophilized ones but only two of them are widely used in clinical practice. Use of different types of meniscal allografts still remains controversial due to preparation method, their biomechanical properties as well as cost which is connected with processing and storage. The main aim of this review is to present the current status of knowledge concerning meniscal allograft preservation and sterilization, especially the advantages and disadvantages of each method. Authors wanted to show a broad spectrum of methods used and conceptions presented by other authors. The second aim is to gather available information about meniscal preservation and sterilization methods in one paper. Deep-frozen and cryopreserved meniscal allografts are the most frequently used ones in the clinical practice. The use of fresh grafts stays controversial but also has many followers. Lyophilized grafts in turn are not applied at present due to some serious drawbacks including reduction of tensile strength, poor rehydration, graft shrinkage and post-transplantation joint effusion as well as increased risk of meniscal size reduction. An application of sterilizing agents make the meniscal allograft free from the bacteria and viruses, but also it may cause serious structure changes. Therefore, choosing just one ideal method of meniscal allograft preservation and sterilization is complicated and should be based on broad knowledge and experience of surgeon performing the transplantation.

## Introduction

The meniscus plays a very important role in normal knee function. Menisci are small, crescent-shaped collagen structures between the femur and tibia, consisting of fibrocartilage. Menisci cover the peripheral two-thirds of the articular surfaces of the tibial plateau (Mi Lee and Fu 2000). They are responsible for shock absorption, joint stability, lubrication and congruity as well as load distribution and knee stabilization (Levy et al. 1982, 1989; Henning and Lynch 1985). Lack of the meniscus implies a decrease in surface contact area with a consecutive increase in contact pressure, resulting in gradual disappearance of cartilage within a decade (Shelton 2007; Schubert et al. 2010). Some studies have shown a high risk of knee arthrosis after meniscectomy (Hommen et al. 2007). Joint degeneration following complete meniscus deletion has been documented and recognized as a major cause of osteoarthritis (Johnson et al. 1974; Allen et al. 1984; McNicholas et al. 2000). An alternative to total meniscus deficiency is allograft transplantation. The most frequent indication for meniscus transplantation is persistent pain in the meniscectomized knee. The graft intended for transplantation should fulfill some criteria to be suitable for the patient i.e. it must be of appropriate size and should have good biomechanical properties (McNickle et al. 2009). Nowadays, there are a few types of grafts which can be used for transplantation, but only some of them are widely used in clinical practice.

There are two very important issues to be considered before the transplantation: whether the graft has to contain viable, metabolizing cells that are able to divide, and—on the other hand—whether it has to keep its scaffold architecture to function rightly (Gelber et al. 2009). Apart from these questions, surgeons performing meniscal transplantation should be aware of the advantages and disadvantages of meniscal allograft preservation and sterilization methods, because the way of the allograft pre-transplantation preparation of the allograft can affect its physical properties and strength. As far as the preservation methods are concerned, four types of meniscal allografts are distinguished: fresh, cryopreserved, deep-frozen and lyophilized ones (von Lewinski and Wirth 2010).

The aim of this review is to present the current status of knowledge concerning meniscal allograft preservation and sterilization, especially the advantages and disadvantages of each method.

## Search strategy and eligibility criteria

At least two of the authors searched independently the bibliographic databases such as SpringerLink, Science Direct, Wiley Online Library and PubMed in order to find essential articles on meniscal allograft preservation and sterilization methods. To obtain only the preferable articles from all the papers on menisci, the authors used the following key words: “meniscal preservation” and “meniscal sterilization” alone as well as in combination with “cryopreserved”, “deep-frozen”, “fresh allograft”, “lyophilized”, “irradiated” and “ethylene-dioxide sterilization” (in various combinations, using Boolean operators AND and OR). After the first review of the chosen articles, those with some of the key words in title were classified for further analysis. In the next step, also articles without the key words in the title but related to meniscal allograft preparation were assessed. Following this preliminary evaluation, the abstracts of the selected articles were reviewed and if the subject matter of the chosen articles coincided with the topic of the study, the article was read as a whole.

To identify additional important studies missed in the process of original literature search, the bibliographies of the articles were also reviewed. Finally, the review papers and books were also evaluated to check their bibliographies and find there other original works.

The authors’ objective was to find a large number of publications on meniscal allograft preparation to show a broad spectrum of the methods used and conceptions presented by others authors. Therefore the year of publication had a secondary importance.

One of the issues raised in the articles involved using one or more of the types of grafts (fresh, cryopreserved, deep-frozen, lyophilized, irradiated, nonirradiated) in human or animal studies: there were studies in which cell migration or collagen net changes were observed after one of the preservation/sterilization methods had been used, as well as comparative studies in which a few methods were described.

According to an additional criterion, the articles taken into consideration were written in English only.

Most of articles found were original works and follow up studies. Some of review articles touching the problem of meniscal preservation methods were also taken into account as a support of this paper. Among 53 references 15 concerned fresh allograft, in 17 papers problem of deep-frozen meniscal allograft was raised and in 18 studies cryopreserved menisci were referred. Lyophilized meniscal allografts were mentioned in 9 reviewed articles.

For better visualization and understanding of the similarities and differences between individual preservation and sterilization methods, the data collected were recorded in MS Excel. The following parameters were taken into account: type of the method, viability of cells, immunogenicity, risk of disease transmission, changes in collagen structure and/or the transplant’s strength as well as storage and logistic problems.

## Types of grafts

### Fresh grafts

Fresh menisci are used for viable meniscal allografting. This type of graft is supported by the viable cells theory which says that fresh tissue contains a large number of these cells, which may have influence on the maintenance of extracellular matrix properties (Verdonk 1997; Fabbriciani et al. 1997; von Lewinski and Wirth 2010). The main advantage of fresh grafts is providing undamaged cells and also keeping cells viable by producing proteoglycans and collagen fiber structures. It is important due to a significant role of proteoglycans in meniscal structure. They have the ability to bind water and can affect physical properties of the meniscus (Gelber et al. 2012).

To keep the best possible fresh meniscal allografts properties, a few restrictions must be respected. To maintain the maximal viability and metabolic activity of the meniscal cells, procurement should take place as soon as possible and not longer than 12 h after death (Schubert et al. 2010). Other sources suggest, that removal and grafting should be carried out within 4–6 h in order to maintain the cell viability of the graft (Jackson and Simon 1992).

The procedure of donation should be performed as follows: after harvesting under sterile conditions, the graft must be transported in a sterile saline solution. In the next step, the graft should be placed in a culture medium containing 20 % of the recipient’s serum and stored at 37 °C in continuously controlled environmental conditions. The parameters such as viability of cells in the fresh graft must be carefully documented (Verdonk 2002; Verdonk et al. 2006; Schubert et al. 2010).

Viable meniscal transplantation is sometimes criticized as a technique which is quite expensive and logistically demanding in view of a quite short period of time between the donor’s death and transplantation. Other authors reported that the viability of the donor cells is not so important because host cells can repopulate the graft within a few weeks after transplantation (Jackson et al. 1993).

It is worth remembering that the use of fresh tissue as a transplants is always associated with a high risk of disease transmission. Also in case of fresh meniscal allografts transplantation, there is a risk of transmission of pathogens and requirements to perform special tests which can exclude infection (Verdonk and Kohn 1999). Instant transplantation is especially associated with a high risk of disease transmission. However, Polish scientists proved that freshly collected menisci can be stored for 14 days under controlled conditions without a significant loss of cell viability (Kaźnica et al. 2009). Nevertheless, fresh meniscal allografts are not transplanted in Poland.

### Cryopreserved grafts

Progress in low-temperature biology has produced high-viability preservation for cells and tissues. Cryopreserved meniscal allografts are menisci that are submerged in a solution with a cryoprotective agent, a culture medium and an antiseptic agent. When the impregnation is completed, the graft is slowly frozen under controlled conditions (paying special attention to the temperature and speed of freezing) to minimize cellular tears generated during the freezing process. This type of grafts is stored at −196 °C. Theoretically, cryopreservation may protect viable donor cells due to the use of a cryoprotectant such as glycerol or dimethyl sulfoxide, but even if the cryopreserved graft still contains viable cells after thawing, their longterm survival remains controversial (Fabbriciani et al. 1997; Verdonk and Kohn 1999; Schubert et al. 2010). Dimethyl sulfoxide and glycerol protect cells against the formation of intracellular ice crystals. According to recent data, the percentage of cell survival after cryopreservation has been established between 4 and 54 % (Gelber et al. 2009). Other data indicate a percentage of viable cells after thawing between 10 and 40 % (Milton et al. 1990; Jackson and Simon 1992).

Cryopreservation is a difficult and costly technique, and it may increase the risk of transmission of infectious diseases (Fabbriciani et al. 1997). In terms of biomechanics, this technique does not seem to alter the microarchitecture or the material properties of the meniscus (Gelber et al. 2009). On the other hand, however cumulative evidence suggests that cryopreserved menisci suffer various tissue and metabolic changes as well as some loss of structural details of the cells (Pegg et al. 2006; Villalba et al. 2012). Cryopreservation has its advantages e.g. it allows prolonged allograft storage, but as a technique it is rather problematic (Binnet et al. 2012). Some authors predicate that cryopreservation worked well in clinical and experimental studies, but no significant differences could be found in comparison to deep-frozen techniques (Fabbriciani et al. 1997; von Lewinski and Wirth 2010).

### Deep-frozen grafts (fresh-frozen)

Deep freezing of the meniscal allograft at −80 °C is one of the most common preservation methods of the meniscus. This method is technically simple and minimally immunogenic. The menisci harvested under sterile conditions are put into physiologic saline with an antibiotic agent (usually rifampicin) and stored in a deep-frozen state after having been frozen at a fast rate (Ochi et al. 1995). The same graft conservation techniques differ in the procedure description in various studies. Some authors described the deep freezing process as a sudden temperature decrease, brought down within 1 min with the help of liquid nitrogen either to −80 or to −196 °C (Arnoczky et al. 1992; Wada et al. 1998). Others simply freeze samples without processing either at −70 or at −80 °C (Khoury et al. 1994; Fabbriciani et al. 1997; Verdonk and Kohn 1999).

Deep-frozen allografts are easier to store than the fresh grafts, but during the freezing process, donor cells can be destroyed. It may result in denaturation of histocompatibility antigens, which may in turn decrease immunogenicity (Binnet et al. 2012). Afterwards, they are packaged in sterile plastic bags and stored in a mechanical freezer at −80 °C. In the operating theatre, deep-frozen menisci are again soaked in an antibiotic solution, which will be gradually released from the implant for at least 3 weeks after the operation (Schubert et al. 2010).

A very important difference between the deep-frozen and cryopreserved meniscus is that the latter is able to keep some cells viable in view of use a cryoprotectant (Gelber et al. 2008). Furthermore, deep-freezing involves a lower risk of disease transmission, which is possible thanks to the possibility of applying secondary sterilization techniques such as ethylene oxide treatment or gamma irradiation (Arnoczky 1992).

Deep–frozen menisci have also relatively high success rates and they are able to maintenance of biomechanical properties (Sekiya and Ellingson 2006).

**Fig. 1:**
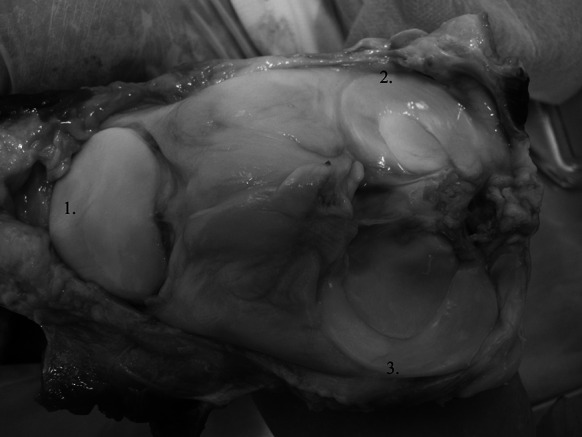
Cadaveric right knee—top view. *1* Patella, *2* lateral meniscus, *3* medial meniscus

**Fig. 2:**
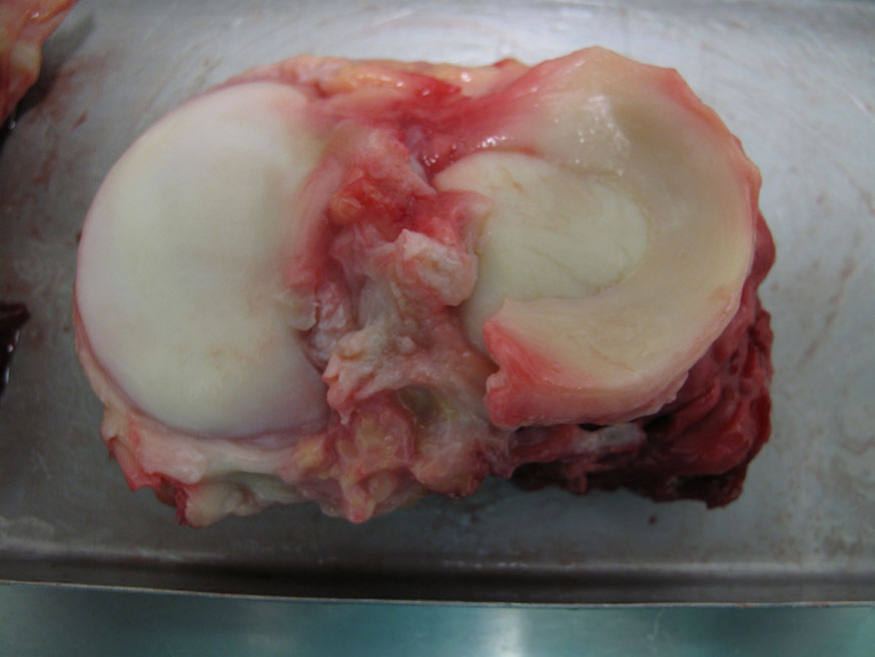
Tibial plateau without medial meniscus

**Fig. 3:**
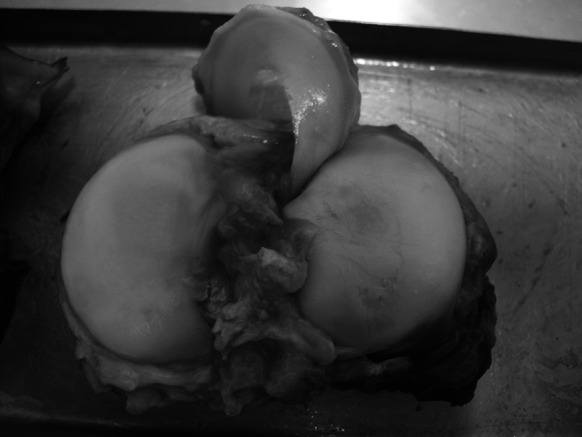
Lateral meniscus fixed with its anterior horn to the tibial plateau

**Fig. 4:**
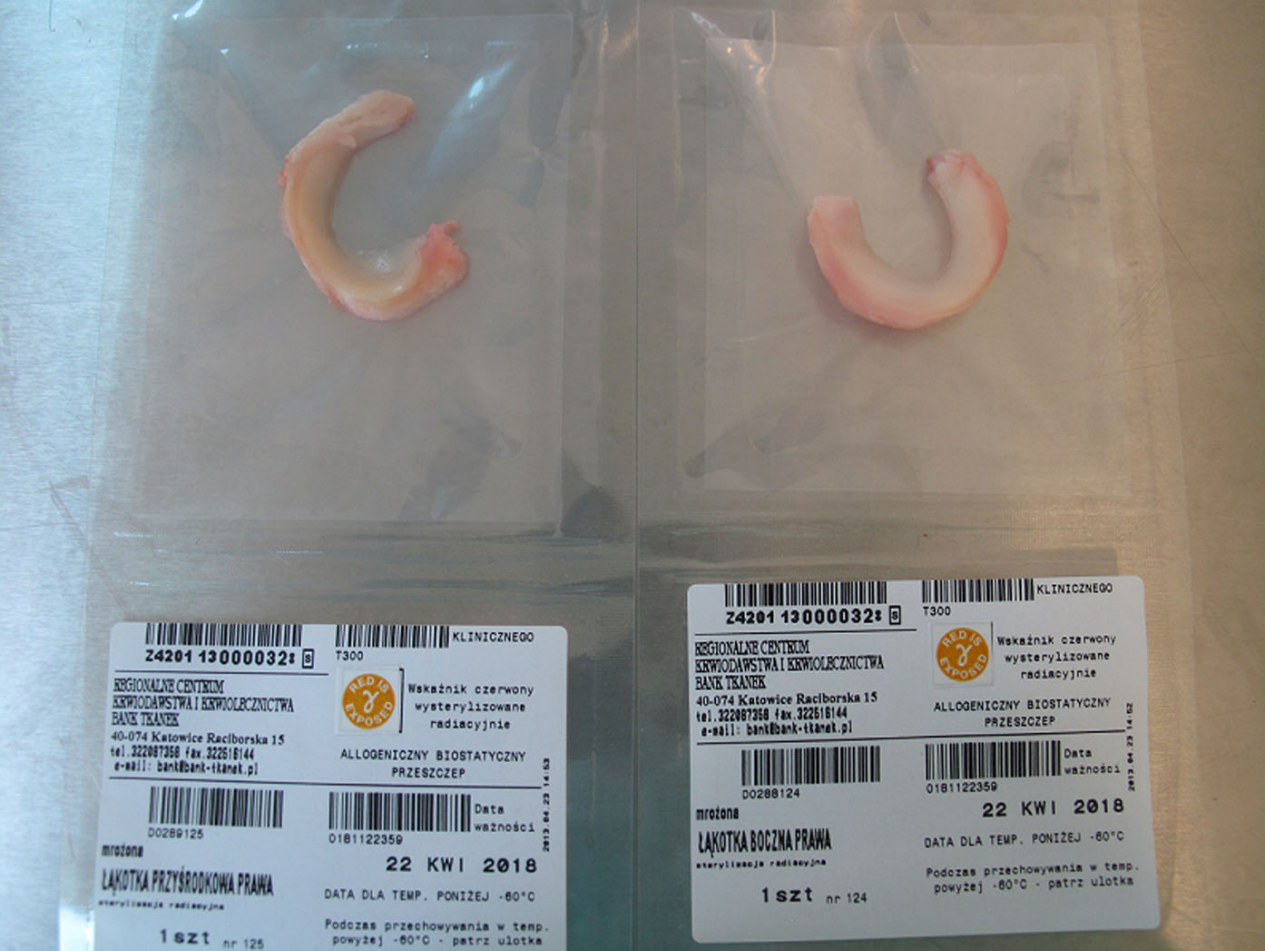
Medial and lateral menisci in plastic bags before γ-irradiation

**Fig. 5:**
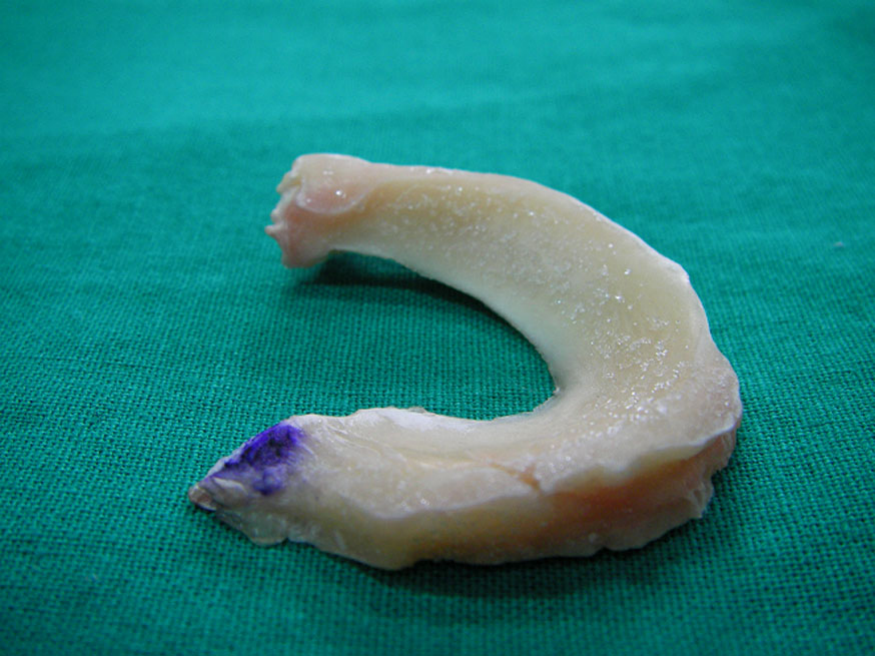
Lateral deep-frozen meniscus in operating theater. Anterior horn is marked

The process of deep-frozen meniscal preparation in Tissue Bank in Katowice, Poland is presented in Figs. 1, 2, 3, 4 and 5.

In Europe the maximal storage period of human deep-frozen tissue is limited to 5 years (Schubert et al. 2010).

### Lyophilized grafts

Lyophilization or freeze-drying, which consists in drying tissue under vacuum and freezing conditions, is an appropriate method to preserve viability of cells if cryoprotective solutions are used (Delloye et al. 1991, 2004). Lyophilization without cryoprotection makes the tissue non-viable and dried. Allografts are thawed and rehydrated before transplantation. Although this method allows for unlimited storage, it also produces changes in the biomechanical properties and size of allografts, which may cause problems with graft sizing during transplantation (Binnet et al. 2012). Freeze-drying is just a preservation method and cannot be treated as a kind of sterilization. Lyophilization is probably the most convenient method as regards storage because dried tissue can be kept at room temperature, but at the same time it is the least common among preservation techniques. Sterilization of lyophilized tissues is troublesome, therefore irradiation at 25 kGy (2.5 Mrad) is usually associated (Yahia et al. 1993; Dziedzic-Goclawska et al. 2005). According to the data collected by some authors in a clinical setting, dried tissue is also irradiated for final sterilization. This combined process of lyophilization and irradiation appears to be detrimental to the tissue, because it results in a deep changes in the physical structure of the extracellular matrix (Delloye et al. 2004). Despite many advantages of lyophilization, this method is not applied at present due to some serious drawbacks including reduction of tensile strength, poor rehydration and graft shrinkage as well as increased risk of meniscal size reduction (Lubowitz et al. 2007; Gelber et al. 2008).

The compilation of the pros and cons of each of the meniscal allograft preservation methods is shown in Table 1.

**Table 1:** Characteristic of meniscal preservation methods

Type of graft	Preparation technique	Cells viability	Immunogenicity	Risk of disease transmission	Storage	Sterilization	Biomechanical properties	Cost	Usage
Fresh	Quite complicated and demanding	Yes	Yes	Potentially high	In incubators, expensive	No	Excellent	Expensive	Rarely
Fresh-frozen	Simple	No	No	Low	at −80 °C	Yes	Controversial	Quite cheap	Often
Cryopreserved	Quite demanding	Yes	Yes	Potentially exist	at −196 °C	No	Controversial	Quite expensive	Quite often
Lyophilized	Complicated	No	No	Low	Unlimited	Yes	Poor	Quite expensive	No longer

## Sterilization methods

Sterilization of the meniscal allograft is performed to reduce the risk of disease transmission. Generally, sterilization may result in killing viable cells and is not performed on fresh and cryopreserved grafts (Lubowitz et al. 2007).

### Gamma irradiation

Gamma irradiation has bactericidal and virucidal properties. It is currently the most common method of sterilizing soft tissue allografts including menisci. Two mechanisms are responsible for creating the virucidal and bactericidal effects of gamma irradiation. One of them, is the direct alteration of nucleic acids leading to genome dysfunction, and the other one—generation of free radicals, mainly from liquid water (Hansen and Shaffer 2001; Vangsness et al. 2003).

To enhance allograft safety, it is recommended that radiation-sterilized tissue grafts be packed in plastic bags made of polymeric materials that are resistant to doses higher than needed for sterilization of tissue grafts and non-reactive with chemical components which can be present in transplant (Dziedzic-Goclawska et al. 2005).

Results indicate the differences in the efficacy of gamma irradiation in the presence and absence of free water, therefore tissue exposed to gamma irradiation in the frozen or freeze-dried state must be treated with significantly higher doses to achieve the same effect as it would be if the item were in the liquid, hydrated state (Vangsness et al. 2003). Studies also have shown that gamma irradiation significantly alters the initial biomechanical characteristic of soft tissue allografts in a dose-dependent manner. Doses as low as 2 Mrad have been shown to reduce the initial stiffness and strength of the tendon allograft. It is unknown whether or not this tear in biomechanical properties has an effect on the clinical result (Rihn et al. 2006). Because of the significant changes in the biomechanical properties of the graft, non-irradiated allografts are generally more frequently used than irradiated ones (Lee et al. 2012).

### Ethylene oxide

The use of ethylene oxide is a type of a chemical sterilization method which is performed with appropriate bactericidal or virucidal solutions (Jackson et al. 1990; Lubowitz et al. 2007).

For more than 40 years ethylene oxide has been used for sterilization of heat- and moisture-sensitive medical devices and tissue. Ethylene oxide is applied in a gaseous state (boiling point, 10.7 °C) in mixture with inert diluents such as CO_2_ to avoid accidents during processing because of its flammability (Vangsness et al. 2003; Dziedzic-Goclawska et al. 2005).

This substance produces a metabolic by-product, ethylene chlorohydrin, which results in a significant cell response and synovial inflammation and therefore it is not recommended as a sterilization agent (Caldwell and Shelton 2005; Binnet et al. 2012).

### Other sterilization methods

The ideal method of sterilization should be safe and easy to use, and it should give very good anti-septic results. Scientists are still looking for an appropriate way of sterilization with good tissue penetration. Among the new methods being developed are supercritical CO_2_ and the use of antioxidants in combination with gamma irradiation (Vangsness et al. 2003). However, in some countries such as Poland, these methods are not commonly used yet.

Some researchers tested application of substances or methods used for bone sterilization in case of soft tissues grafts, but usually without success.

In 2008 Scheffler et al. used peracetic acid (PAA) as a sterilizing agent to investigate its influence on revascularization and recellularization of ACL grafts. PAA has been generally used for bone graft sterilization and did not impair the mechanical properties of soft tissues in studies performed in vitro. However, the results obtained in 2008 has shown PAA sterilization cannot be used for soft tissue allografts because of slowed remodeling activity and reduced mechanical properties of grafts compared to the control groups (Scheffler et al. 2008).

In 2012 in turn, Schmidt et al. (2012) performed experiment using electron beam irradiation (Ebeam) for sterilization of tendons. They investigated influence of Ebeam irradiation on biomechanical properties of free tendon grafts. Ebeam irradiation has few advantages in comparison to standard gamma irradiation: firstly, it can be operated as a fast throughput method which gives more accurately control of sterilization environment and the applied dosage. And secondly, the effectiveness of sterilization is comparable to gamma irradiation (Reid 1998; Seto et al. 2008; Schmidt et al. 2012). Despite this, results of Schmidt’s experiment has shown that high dose of Ebeam irradiation should not be recommended for soft tissue sterilization because of decreased biomechanical properties of grafts treated Ebeam in compared to fresh-frozen grafts without sterilization.

Despite the development of soft tissue graft sterilization methods, gamma irradiation still remains the gold standard in sterilization of these types of soft grafts which can be sterilized.

## Discussion

The ideal meniscal transplant should be safe, i.e. sterile and non-immunogenic, durable and easy to store and transport. However, each of the meniscal preservation and sterilization methods presented in this work has its pros and cons.

Nowadays, the most commonly implanted menisci are of two types: deep-frozen and cryopreserved, but fresh meniscal allograft transplantations has also grown in popularity. According to some of researchers, maintenance of living chondrocytes within the meniscus is required for a successful transplantation. Chondrocytes occupy only about 5 % of the structure, but their role is very significant. They are responsible for the presence of the extensive surrounding matrix that comprises a highly complex network of collagen fibrils, associated proteoglycans and other non-collagenous proteins (Villalba et al. 2012). Thus, it can affect the mechanical integrity of the following transplantation (Lubowitz et al. 2007). In a comparative study involving fresh and cryopreserved grafts, the cells in fresh grafts were filling their lacunae, round in shape with round nuclei, and the apoptotic index in fresh menisci was statistically significantly lower in comparison to cryopreserved grafts (Villalba et al. 2012). It is a very important clue when the major desirable feature is viability of cells. But not all researchers are enthusiasts of the cell viability theory. Some indicate that the time of cell survival is very short and in a goat model it does not exceed 1 month (Jackson et al. 1993). On the other hand, Verdonk et al. (2006) have demonstrated the results of their first 100 meniscal transplantation procedures using fresh allografts. They used 39 medial and 61 lateral menisci. The time of outcomes evaluation was equal a mean of 7.2 years. About 28 % of the medial and 16 % of the lateral allografts failed, which means that persistent pain and/or poor function was occurred.

Other studies show that donor cells are able to remain in the allograft all the time (Lubowitz et al. 2007).

Taking into account broad spectrum of meniscal allograft studies, the question of animal experiments need to be explained. It is necessary to focus on animal studies because most of information regarding meniscis’ biology and function has been obtained throught these types of investigations. Animal studies on menisci are strictly connected with animal models of osteoarthritis. There are few general models used in meniscal studies: ovine, canine and goat but sometimes, smaller animals such as rabbits and rats are also accepted (Bendele 2001). Due to the size of menisci, bigger animals are more suitable for meniscal research but still, animal studies cannot be directly compared with clinical trials. The main reason is animal healing response, which is more robust than humans. Besides, the differences in kinematics of knee joints between quadruped and biped are quite considerable and cannot be compared (Lubowitz et al. 2007).

However, animal studies can be very useful if we want to prove the hypothesis on living organism or in case of testing hypothetical therapies or alternative treatments of menisci before the transplantation.

Study performed by Chevrier et al. including histological and immunohistochemical analysis as well as environmental scanning electron microscopy (ESEM), suggests that the main structural features of the menisci are similar in sheep and human, but significantly different in rabbit (Chevrier et al. 2009). Therefore, some of animal studies can provide valuable information on meniscal features and the knowledge gained from studies in animal models can be very helpful in clinical trials.

An animal study performed by Jackson et al. 1993 on goat model indicates for example that donor cells in a fresh allograft are totally replaced by host cells within a few weeks (Jackson et al. 1993). These data may suggest that viability of donor cells is not as important as some scientists believe, and the graft does not have to contain a large number of them. A natural tendency of the recipient’s cells to penetrate and repopulate the transplant seems to confirm this theory.

Studies performed by Arnoczky et al. (1990) provides some information about graft healing in a canine model. 14 cryopreserved medial meniscal grafts were transplanted into canine knees and the data received 6 months after the operations showed that all the grafts had healed retaining its function and appearance. Histological studies indicate on post-transplantation decrease in cellularity within 2 weeks after operations, but the cellular and metabolic activity in the transplants returned to normal within 3–6 months after the operation. In a very similar study performed by the same author (Arnoczky et al. 1992) deep-frozen menisci also were tested in a canine model. It showed that deep-freezing killed all the cells in the transplant but after 3 months the graft was repopulated by host cells except it central part. Therefore the issue concerning the importance and maintenance of viable donor cells inside the meniscal allograft remains open to discussion. However, comparison of animal and human studies is difficult due to many differences between species and detailed description of all animal models using in meniscal studies requires separate publication.

A very important question concerning meniscal transplantation is recipient’s immune response against the donor cells in some types of allografts. Admittedly, recipients of fresh meniscal allografts do not require immunosuppression, but the importance of the recipient’s immune response to the clinical result remains unknown (Goble et al. 1999). Meniscal allografts have been demonstrated to express Class I and II histocompatibility antigens, which confer the potential for host immune response. The use of bone plugs or bone bridge attached to the meniscal graft may increase the risk of immune response as bone grafts are well known to be immunogenic (Khoury et al. 1994; Lubowitz et al. 2007). To compare the immunogenicity of fresh meniscal allografts, a study in immunosuppressed and normal rats was performed. It demonstrated increased graft survival up to 21 weeks in the immunosuppressed population, whereas in the normal animals, histologic evidence of rejection was noted. It may indicate important contribution of immune system in the transplant survival, even if the immune response is not clearly observable (Wada 1993). However, other studies reported that fresh meniscal allografts in conjunction with osteochondral allografts in humans did not entail any significant immune response at an average follow-up of 4.5 years (Zukor et al. 1990).

The two methods excluding problems with immunogenicity are deep-freezing and lyophilizing but their use may compromise the biomechanical function of the meniscus. Neither of these methods is perfect. Deep-freezing can affect properties of the collagen net inside the meniscus. Gelber et al. (2008) have shown that the freezing process damages the meniscis’ collagen net of the meniscus in terms of both the size and degree of disarray of the collagen fibril. Lyophilization in turn is not applied. Most of the methods used during menisci processing can change the immunogenicity of the graft. It is not always a good modification: for instance, glutaraldehyde processing to decrease immunogenicity is likely to make the meniscus too stiff (Canham and Stanish 1986). Excessive stiffness may induce significant problems during operation. If the allografted meniscus is stiffer than the normal meniscus, the grafted one will cause more friction between the articular surfaces than in the meniscectomized knee and will accelerate articular degeneration and dysfunction. Ideally, a grafted meniscus should be congruous with articular surfaces and have a coefficient of friction and elasticity similar to the normal meniscus (Ochi et al. 1995).

An issue concerning graft transplantation that requires explanation is the risk of disease transmission. The data of 1991 estimate the risk of HIV transmission by frozen connective tissue allografts as 1/8,000,000 (Conway et al. 1991). According to recent data, the risk is from 1 in 173,000 to 1 in 1,000,000 (McAllister et al. 2007). Some sources report that gamma irradiation with at least 3.0 Mrad is necessary to inactivate HIV-1 DNA as determined by testing with PCR, but some of them mention the dose equal to or exceeding 3.6 Mrad to inactivate HIV (Conway et al. 1991). As a result, when irradiation exceeds 3 Mrad, graft sterilization is improved, but this is at the risk of compromising the material properties of the graft (Binnet et al. 2012). However, according to studies of Yahai and Zukor (1994), when irradiation is equal to 2.5 Mrad and above, it is enough to induce mechanical alterations in meniscal allograft.

Given the complexity of the main subject of this paper, the legislation of competent authorities should be taken into account while choosing a meniscal preservation method.

In the Member States of the European Union, proper medical documentation and compliance with strict guidelines are required by Directive 2004/23/EC of the European Parliament (2004) and relevant Commission directives—Commission Directive 2006/17/EC (2006a) as well as Commission Directive 2006/86/EC (2006b).

Any doubts concerning the correct handling of the graft including the ways of preparation of the meniscal allograft, required equipment, graft safety and provision of optimal storage conditions, should be arbitrated taking into account the above-mentioned regulations.

The data presented in this article show that choosing just one ideal method of meniscal allograft preservation and sterilization is complicated and requires great experience and broad knowledge about its biology and function.

It seems that there is no one reliable and unique method to obtain a sterile, safe and viable meniscal allograft which could be useful for a very long time after transplantation. Due to many differences in the results of studies caused by the number of groups, search strategy and various methods of data analysis, further research is necessary to find new ways of graft processing, to make it safer, more durable and keep its viability.

Although, based on literature review and own experience, at present fresh-frozen meniscal allografts seems to be the best alternative for total meniscectomy. Fresh-frozen grafts are simple to preparation and non-immunogenic. Even if this type of meniscal allograft has a little bit worse biomechanical properties than fresh graft, it is safer for patient and it is one of the most widely used in clinical practice.
